# *Brachybacterium paraconglomeratum* Endophthalmitis Postcataract Operation

**DOI:** 10.1155/2020/1513069

**Published:** 2020-03-12

**Authors:** Kazuhiro Murata, Kenji Ozawa, Hideaki Kawakami, Kiyofumi Mochizuki, Kiyofumi Ohkusu

**Affiliations:** ^1^Department of Ophthalmology, Gifu University Graduate School of Medicine, 1-1 Yanagido, Gifu-shi, Gifu 501-1194, Japan; ^2^Department of Ophthalmology, Gifu Municipal Hospital, 7-1 Kashima-cho, Gifu-shi, Gifu 500-8513, Japan; ^3^Department of Microbiology, Tokyo Medical University, Japan

## Abstract

**Purpose:**

To present our findings in a case of delayed-onset postoperative endophthalmitis caused by *Brachybacterium paraconglomeratum*, a rare organism. *Observations*. A 57-year-old man presented with epiphora and photophobia 17 weeks after a routine cataract extraction with phacoemulsification and intraocular lens implantation. Because endophthalmitis was suspected to be caused by a low-grade pathogen or fungus, an anterior chamber tap was performed. However, both multiplex PCR and culturing were negative. The patient was treated with topical cefmenoxime, oral minocycline, and subconjunctival injection of vancomycin and ceftazidime, but the intraocular inflammation increased. Then, the anterior chamber was tapped again, and the second PCR amplification and direct sequencing which targeted *Actinomycetes* detected *Brachybacterium paraconglomeratum*, a rare organism.

**Conclusion:**

This is the first reported case of an ocular disorder caused by *B. paraconglomeratum*. We recommend that *B. paraconglomeratum* be considered in cases of delayed-onset postcataract endophthalmitis.*B. paraconglomeratum*. We recommend that *B. paraconglomeratum* be considered in cases of delayed-onset postcataract endophthalmitis.

## 1. Introduction

A rapid diagnosis is important for a fast recovery from ocular diseases and maintenance of ocular function. However, it is occasionally difficult to detect the causative pathogens from the collected samples by conventional examinations, microscopy, and culturing. The advances in the molecular techniques for detecting and identifying organisms by the polymerase chain reaction (PCR) method have enabled more accurate and faster identification of various pathogens which were unable to be detected earlier [1, 2].

We report our findings in a case of ocular infection caused by *B. paraconglomeratum* that was detected by PCR.

## 2. Case Report

**Figure 1:**
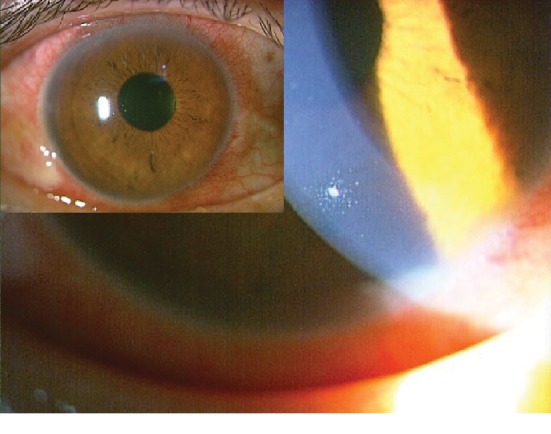
Photographs (insert: fluorescent staining) before the second anterior chamber examination. His right eye has conjunctival hyperemia, fine keratic precipitates, and inflammatory cells in the anterior chamber.

A 57-year-old Japanese man underwent routine cataract surgery. He was examined in our hospital 17 weeks postoperatively with a one-week history of epiphora and photophobia in his right eye. Our examination showed that his decimal best-corrected visual acuity (BCVA) was 1.2 and his intraocular pressures was 19 mmHg bilaterally. His right eye had conjunctival hyperemia, fine keratic precipitates, and grade 1+ inflammatory cells in the anterior chamber. The vitreous and retina had no lesions bilaterally. His general health was good from birth. An initial diagnosis of iritis was made, and he underwent steroid therapy with the methods of eye drops and subconjunctival injection. The symptoms in his right eye improved only slightly. However, he had eye pain and recurrent hyperemia 3 days later. Because postoperative bacterial endophthalmitis without inflammation in the vitreous was suspected, the anterior chamber was tapped. He received repeated subconjunctival injections of vancomycin 5 mg/0.5 ml and ceftazidime 11.25 mg/0.5 ml, as well as topical cefmenoxime and 200 mg of oral minocycline. The condition in the right eye deteriorated still more, and the results of both culture and multiplex PCR examination (outside laboratory) were negative. Considering the possibility of low-grade bacterial or fungal infection, the anterior chamber was tapped a second time (Figure 1). The second PCR examination at the Tokyo Medical University targeted Actinomycetales and whole fungus. The PCR amplification and direct sequencing showed that *B. paraconglomeratum* was present in the aqueous.

Treatment was initiated with topical cefmenoxime and betamethasone, 750 mg/day of oral amoxicillin, and erythromycin ointment. The number of cells in the anterior chamber gradually decreased, and the patient had no signs of inflammation in the anterior chamber over the next 8 months. The final BCVA was 1.2 bilaterally.

## 3. Discussion

*Brachybacterium* has been isolated from poultry deep litter by Collins et al. in 1988 [3]. The *Brachybacterium* spp. belong to the Actinomycetales and are nonsporing gram-positive rods [3]. They are considered to be nonharmful environmental bacteria [1]. The *Brachybacterium* spp. are presently confirmed to be made up of 20 strains [4].

*B. paraconglomeratum* was first reported by Takeuchi et al. [5]. The colonies were circular, entire, low convex, smooth, opaque, and pale brown [5]. *B. paraconglomeratum* has a slower growth rate in culture than other *Brachybacterium* spp. [5].

A human infection related to *Brachybacterium* spp. was first proposed by Tamai et al. in 2018, but there are only a few reports of *Brachybacterium* spp. isolated from human samples [1, 6–8]. It is not known why human infections by *Brachybacterium* spp. are rare.

Brain-Heart Infusion (BHI) agar is recommended as a universal medium for aerobic bacteria and for the primary recovered fungi and Actinomycetales [9, 10]. Takeuchi et al. also performed a taxonomic study including *B. paraconglomeratum* with BHI agar [5]. However, the laboratories of many hospitals and outside institutions generally use 5% sheep blood agar, chocolate agar, and Sabouraud dextrose agar as conventional agars [1].

Tamai et al. reported that the results of culture and PCR examinations of blood samples were positive for *B. paraconglomeratum*, but cultures of samples of the intervertebral disc were negative for *B. paraconglomeratum* in cultures and microscopic examinations [1]. Our sample from the anterior chamber tap was very small and was negative in culture and PCR examination.

Considering these circumstances, *Brachybacterium* spp. may need more suitable environment for growth than other microorganisms. For example, one may be enriched by nutrition agar and/or other many colleagues, although it is unclear that perhaps *Brachybacterium* spp. may have characteristics of quorum sensing. Culturing of small volume samples of presumed low-grade pathogen should have been performed with BHI agar having high nutritive value.

The advancements of the PCR method has opened the way for further development of the diagnosis level to human infections. However, the detection rate of the whole bacteria and fungi with multiplex PCR examinations is approximately 60 to 80% and not 100% [11]. A rare microorganism or small sample volume can lead to negative results even by PCR examinations. In our patient, the first multiplex PCR examination was unable to detect a pathogen. The primer used was unsuitable to the pathogen. Moreover, the sample volume might be too small after being distributed between the PCR examination and culture. Considering these factors, the sample from the second anterior chamber tap was used for PCR examination only. In addition, the second PCR examination targeted the whole fungi and Actinomycetales referring to the previous reports related to delayed-onset postoperative endophthalmitis [12]. As a result, the pathogen was identified as *B. paraconglomeratum*.

*Brachybacterium* spp. were reported to be susceptible to ampicillin, cefazolin, vancomycin, erythromycin, and rifampicin, and the susceptibilities to gentamicin, clindamycin, and tetracycline varied among the strains. [1, 13–16]. On the other hand, antimicrobial susceptibility of *B. paraconglomeratum* is unknown. Moreover, the antimicrobial susceptibility of our specimen could not be determined because of the negative culture. Our patient was initially treated with oral minocycline but had no sign of improvement. Therefore, after identifying *B. paraconglomeratum*, oral amoxicillin was administered as reported by Tamai et al. [1]. Then, the inflammation in the anterior chamber gradually improved with no recurrences.

Souli et al. reported that the peak level of vancomycin in the aqueous humor was 24.82 ± 3.55 *μ*g/ml at 5 h after a subconjunctival administration [17]. Tamai et al. reported that minimum inhibitory concentration of the isolated *Brachybacterium* spp. was 0.5 *μ*g/ml [1]. Our patient had two subconjunctival injection with vancomycin; however, his eye condition did not improve. Although the reason of deterioration remained to be resolved, the microorganism *B. paraconglomeratum* might have been resistant to vancomycin.

In conclusion, *B. paraconglomeratum* should be considered in the differential diagnosis of organisms related to delayed postcataract endophthalmitis. Further accumulation of cases is needed to provide appropriate treatment to *Brachybacterium* sp. infections in humans.
